# On the Emerging Role of the Taste Receptor Type 1 (T1R) Family of Nutrient-Sensors in the Musculoskeletal System

**DOI:** 10.3390/molecules22030469

**Published:** 2017-03-15

**Authors:** Shoichiro Kokabu, Jonathan W. Lowery, Takashi Toyono, Tsuyoshi Sato, Tetsuya Yoda

**Affiliations:** 1 Department of Oral and Maxillofacial Surgery, Faculty of Medicine, Saitama Medical University, Moroyama-machi, Iruma-gun, Saitama 350-0495, Japan; tsato@saitama-med.ac.jp (T.S.); yoda@saitama-med.ac.jp (T.Y.); 2 Division of Molecular Signaling and Biochemistry, Department of Health Promotion, Kyushu Dental University, Kokurakita-ku, Kitakyushu, Fukuoka 803-8580, Japan; 3 Division of Biomedical Science, Marian University College of Osteopathic Medicine, 3200 Cold Spring Rd., Indianapolis, IN 46222, USA; jlowery@marian.edu; 4 Bone & Mineral Research Group, Marian University College of Osteopathic Medicine, 3200 Cold Spring Rd., Indianapolis, IN 46222, USA; 5 Division of Anatomy, Department of Health Promotion, Kyushu Dental University, Kokurakita-ku, Kitakyushu, Fukuoka 803-8580, Japan; toyono@kyu-dent.ac.jp

**Keywords:** taste receptor, bone, skeletal muscle, sarcopenia, osteoporosis, T1R3, myogenesis, bone remodeling

## Abstract

The special sense of taste guides and guards food intake and is essential for body maintenance. Salty and sour tastes are sensed via ion channels or gated ion channels while G protein-coupled receptors (GPCRs) of the taste receptor type 1 (T1R) family sense sweet and umami tastes and GPCRs of the taste receptor type 2 (T2R) family sense bitter tastes. T1R and T2R receptors share similar downstream signaling pathways that result in the stimulation of phospholipase-C-β2. The T1R family includes three members that form heterodimeric complexes to recognize either amino acids or sweet molecules such as glucose. Although these functions were originally described in gustatory tissue, T1R family members are expressed in numerous non-gustatory tissues and are now viewed as nutrient sensors that play important roles in monitoring global glucose and amino acid status. Here, we highlight emerging evidence detailing the function of T1R family members in the musculoskeletal system and review these findings in the context of the musculoskeletal diseases sarcopenia and osteoporosis, which are major public health problems among the elderly that affect locomotion, activities of daily living, and quality of life. These studies raise the possibility that T1R family member function may be modulated for therapeutic benefit.

## 1. Introduction

The special sense of taste acts as the guardian and guide for food intake and is essential for body maintenance [1]. The sensation of taste can be divided into five distinct qualities; salty, sour, bitter, sweet, and umami [2]. The sensation of bitter and sour tastes deters us from ingesting potential toxic substances and strong acids while sweet, umami, and salty tastes encourage us to eat foods containing carbohydrates, amino acids, and sodium, respectively [2]. Salty and sour tastes are transduced via ion channels or gated ion channels which are expressed in gustatory tissues and in a variety of non-gustatory tissues such as the kidney [3]. In contrast, sensing of the remaining tastes relies on two distinct families of G protein–coupled receptors (GPCRs) named the taste receptor type 1 (T1R) family, which senses sweet and umami, and the taste receptor type 2 (T2R) family, which senses bitter [4,5,6,7,8].

Although the T1R and T2R receptor families drive different taste sensations, they share similar downstream signaling pathways wherein receptors are coupled to a heterotrimeric G protein consisting of α-, β- and γ-subunits. Activation of the T1R or T2R triggers the separation of the α-subunit, which is commonly α-gustducin, from the β/γ subunits. Downstream events include stimulating phospholipase C β2 (PLCβ2) and second messengers that consequently increase cytosolic Ca^2+^ levels to activate the transient receptor potential cation channel M5 (TRPM5) and induce membrane depolarization and action potential generation [9,10].

The T1R family includes three members, T1R1, T1R2, and T1R3, which form heterodimeric complexes that exhibit differential recognition of ligands: T1R3 complexes with T1R1 to form the umami taste receptor, which responds to amino acids, while T1R3 complexes with T1R2 to form the sweet taste receptor, which responds to molecules such as glucose [7,8,9,11]. Although these functions were originally described in and most commonly assigned to gustatory tissue, the first indication that T1Rs and elements of the taste transduction cascade might exist outside of the mouth was more than 20 years ago when the expression of the taste signaling–associated G protein α-gustducin was observed in brush cells of the stomach and intestine [12]. Subsequent studies have demonstrated the expression of and a functional role for T1R family members in a wide variety of tissues and cell types such the pancreatic β-cells [13], adipose tissue [14], heart [15], and central nervous system [16]. Thus, despite the name “taste receptor”, these GPCRs are now viewed as “nutrient sensors” that play important roles in chemoreception to detect global glucose and amino acid status [17]. 

The function of T1R family members in several non-gustatory systems has been summarized previously [2,18]. However, we are particularly struck by several recent reports highlighting T1R family members in the musculoskeletal system [19,20,21,22] and wish to briefly review these findings in the context of the musculoskeletal diseases sarcopenia and osteoporosis, which are major public health problems among the elderly that affect locomotion, activities of daily living, and quality of life [23]. 

## 2. Sarcopenia and the Role of the T1R Family in Myogenesis

Sarcopenia is characterized by an age-related loss of skeletal muscle mass and a decline in muscle strength that compromises the health span [24]. The prevalence of sarcopenia is approximately 10% among adults aged 60 or older [25], resulting in a cost over US$18 billion in 2001 alone [26]. Thus, there is an unmet and urgent need for strategies that will improve skeletal muscle mass and/or function in aging adults.

Satellite cells are skeletal muscle stem cells that provide a regenerative capacity for skeletal muscle. Aged skeletal muscle shows a profound regenerative impairment that contributes to physical incapacitation. Aged skeletal muscles fail to retain stem cell quiescence [27,28,29] and both the number and the functionality of muscle stem cells decline with aging [27,28,29,30,31,32]. The process of autophagy, which involves the degradation of long-lived proteins and damaged organelles in lysosomes, has been implicated in the aging of different model organisms [29,33,34,35,36]. Recent work indicates that autophagy maintains the multipotency of satellite cells by preventing senescence [37], indicating that the appropriate regulation of autophagy is required for the maintenance of skeletal muscle mass and function. This also raises the possibility that dysregulation of autophagy is involved in the pathogenesis of sarcopenia.

Differentiation of skeletal muscle fibers—myogenesis—is a complex and tightly regulated process involving the commitment of mesenchymal stem cells to the myogenic lineage, myoblast proliferation, and exit from the cell cycle. This multi-step process is orchestrated by a handful of genes encoding the myogenic regulatory factors (MRFs), which belong to the basic-helix-loop-helix (bHLH) transcription factor family and consist of Myf5, MyoD, MRF4, and Myogenin. Although genetic models have shown that MRFs display a certain degree of functional redundancy [38,39], Myf5 and MyoD are responsible for myoblast lineage commitment, regulating the formation, proliferation, and longevity of myoblasts; MRF4 and Myogenin instead play critical roles in the regulation of the final stage of differentiation [40,41,42].

We recently reported that MRFs regulate the expression of the bi-functional T1R family member T1R3: overexpression of MyoD and Myogenin induces murine *T1R3* promoter activity and ChIP analysis demonstrated that MyoD and Myogenin bind to the endogenous murine *T1R3* promoter region and increase mRNA levels of endogenous *T1R3* in the murine mesenchymal stem cell line C3H10T1/2, which has myogenic potential [20]. Comparative and functional genomic results examining transcription factor binding sites (Table 1) raise the possibility that the *cis*-regulatory elements by which MRFs regulate T1R3 expression are conserved across species [20]. These observations are consistent with the fact that expression levels of T1R3 increase with myogenic differentiation of the murine myoblast cell line C2C12 and skeletal muscle cell lines [20]; unpublished data from our group indicates that *T1R1* expression also correlates with advanced differentiation during myogenesis (data not shown). These findings are supported by the findings that T1R1 and T1R3 are endogenously expressed in the skeletal muscle tissue and that skeletal muscle cells of *T1R3* knockout mice exhibit decreasing activity of mechanistic target of rapamycin complex 1 (MTORC1) and a higher frequency of autophagy [21,43], suggesting that T1R3 function is critical to detecting nutrient status since skeletal muscle is the main source of stored amino acid during times of amino acid deprivation [44]. For these reasons, we hypothesize that reduced signaling through T1R3 is involved in the pathogenesis of skeletal muscle diseases such as sarcopenia through improper regulation of autophagy.

## 3. Osteoporosis and the Role of the T1R Family in Postnatal Bone Remodeling

Osteoporosis is a skeletal disease characterized by low bone mass and microarchitectural deterioration of bone tissue with a consequent increase in bone fragility and susceptibility to fracture [45]. In 2010, more than 10 million Americans over the age of 50 had osteoporosis, with another 43 million Americans at risk for the disease [46]. It is estimated that greater than 1.5 million fragility fractures occur each year, with an annual health care cost of at least US$14 billion [47] and, by 2025, the health care expenditures for osteoporotic fractures will approach US$25.3 billion. The pathogenesis of osteoporosis is complex, with numerous pathways implicated as playing a role in regulating bone mass [48]; that said, there is an urgent and unmet need to identify new treatment targets and strategies for improving bone mass and strength in the aging skeleton. 

The T1R family members T1R2 and T1R3, which heterodimerize to form the sweet taste receptor, were recently identified as playing a role in the regulation of postnatal bone mass. Global loss of either T1R2 or T1R3 expression leads to increased cortical bone mass and trabecular remodeling under high fat conditions with little to no change in glucose tolerance, insulin sensitivity and energy balance [19]. Reduced bone marrow adiposity was observed in these mice and, since bone-forming osteoblasts and marrow adipocytes are both derived from bone marrow stromal cell (BMSC)s, these authors postulated that T1R2 and/or T1R3 regulates cell fate determination in the bone marrow microenvironment. These findings may hold insight into human disease as the balance between adipocyte and osteoblast differentiation is disrupted in some conditions such that adipocyte differentiation is increased relative to osteoblast differentiation, leading to a reduction in bone mass, increased bone fragility, and an increased susceptibility to fracture [49]. The role of T1R family members in bone formation requires further examination through the BMSC-specific deletion of T1R2 and/or T1R3 mice; however, conditional alleles are not available at present. Furthermore, it is possible that T1R family members may regulate bone remodeling via additional mechanisms since bone resorption is markedly reduced and cortical bone mass is increased in *T1R3* knockout mice under standard dietary conditions [49].

## 4. Conclusions

**Figure 1:**
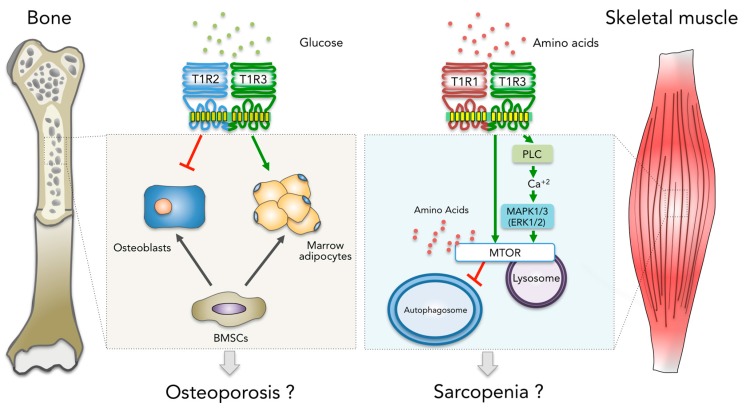
Model for the possible role of T1R family members in the bone marrow and skeletal muscle microenvironments. T1R2-T1R3 detects glucose and regulates cell fate determination of bone marrow stromal cells (BMSCs), promoting adipocyte (AD) differentiation over osteoblast (OB) differentiation. In contrast, T1R1-T1R3 detects amino acids leading to the activation of MTORC1 and inhibition of autophagy, in part through the activation of phospholipase C (PLC), increasing intracellular calcium and the activation of MAPK1-MAPK3. T1R1-T1R3 is required for the amino acid–induced localization of MTOR to the lysosome, which is a necessary step in MTORC1 activation [50].

Recent evidence indicates that T1R family members participate in the regulation of numerous physiological processes in a wide variety of organ systems [18]. In this brief review, we highlighted the emerging evidence that T1R family members are members of the endogenous mechanisms that impact the musculoskeletal system (Figure 1). The studies discussed here raise the possibility that T1R family member function may be modulated for therapeutic benefit.

## Figures and Tables

**Table 1:** Transcription factor binding sites are conserved between *H. sapiens* and *M. musculus* promoter region. Common transcription factor binding sites upstream of *T1R3* were identified using rVista 2.0 for *H. sapiens* and *M. musculus* [20].

No.	Transcription Factor (Description)	No.	Transcription Factor (Description)	
1	AP2 (activator protein 2)	22	LXR
2	AP2α (activator protein 2α)	23	MAZ
3	AP4 (activator protein 4)	24	MYOD (myoblast determining factor)
4	ATF4 (activating transcription factor 4)	25	MYOGENIN
5	CEBP (CCAAT/enhancer binding protein)	26	NGFIC
6	CHCH (Churchill)	27	R (Epstein-Barr virus transcription factor R)
7	CP2	28	RFX (X-box binding protein RFX)
8	CREB (cAMP-response element-binding protein)	29	SEF1
9	DEAF1	30	SMAD
10	E12	31	SMAD4
11	E2A	32	SP1
12	E2F	33	SPZ1
13	E2F1	34	SREBP1
14	EBOX	35	SRF (serum response factor)
15	EGR	36	SRY (sex-determining region Y gene product)
16	FOXO4 (fork head box O4)	37	STAF (Se-Cys tRNA gene transcription activating factor)
17	HEB	38	STRA13
18	HEN1	39	TEF1
19	HSF1 (heat shock factor 1)	40	UF1H3β
20	KROX	41	USF (upstream stimulating factor)
21	LBP1	42	ZID (zinc finger with interaction domain)
